# Soluble Periostin is a potential surveillance biomarker for early and long-term response to chemotherapy in advanced breast cancer

**DOI:** 10.1186/s12935-024-03298-1

**Published:** 2024-03-19

**Authors:** Li Jia, Wenwei Hu, Xu Yan, Jie Shao, Yuhong Guo, Aimin Zhang, Lianzi Yu, Yunli Zhou, Yueguo Li, Li Ren, Dong Dong

**Affiliations:** 1 Department of Laboratory, Tianjin Medical University Cancer Institute and Hospital, National Clinical Research Center for Cancer; Tianjin’s Clinical Research Center for Cancer; Key Laboratory of Cancer Prevention and Therapy, Tianjin; Key Laboratory of Breast Cancer Prevention and Therapy, Tianjin Medical University, Ministry of Education, Huanhuxi Road, Hexi District, Tianjin, 300060 PR China; 2 Department of Gastroenterology, Chinese People’s Liberation Army Rocket Force Characteristic Medical Center, Beijing, 100088 PR China; 3 Department of Anesthesiology, Chinese People’s Liberation Army Rocket Force Characteristic Medical Center, Beijing, 100088 PR China; 4 Department of Pathology, Tianjin Medical University Cancer Institute and Hospital, National Clinical Research Center for Cancer; Tianjin’s Clinical Research Center for Cancer; Key Laboratory of Cancer Prevention and Therapy, Tianjin; Key Laboratory of Breast Cancer Prevention and Therapy, Tianjin Medical University, Ministry of Education, Tianjin, 300060 PR China

**Keywords:** Soluble Periostin, Surveillance, Biomarker, Chemotherapy, Advanced breast cancer

## Abstract

**Background:**

Noninvasive biomarkers for the assessment of response to chemotherapy in advanced breast cancer (BCa) are essential for optimized therapeutic decision-making. We evaluated the potential of soluble Periostin (POSTN) in circulation as a novel biomarker for chemotherapy efficacy monitoring.

**Methods:**

Two hundred and thirty-one patients with different stages of BCa were included. Of those patients, 58 patients with inoperable metastatic disease receiving HER2-targeted or non-targeted chemotherapy were enrolled to assess the performances of markers in recapitulating the chemotherapy efficacy assessed by imaging. POSTN, together with CA153 or CEA at different time points (C0, C2, and C4) were determined.

**Results:**

POSTN levels were significantly associated with tumor volume (*P* < 0.0001) and TNM stages (*P* < 0.0001) of BCa. For early monitoring, dynamics of POSTN could recapitulate the chemotherapy efficacy among all molecular subtypes (Cohen’s weighted kappa = 0.638, *P* < 0.0001), much better than that of carcinoembryonic antigen (CEA) and cancer antigen 153 (CA15-3). For early partial response, superior performance of POSTN was observed (Cohen’s weighted kappa = 0.827, *P* < 0.0001) in cases with baseline levels above 17.19 ng/mL. For long-term monitoring, the POSTN response was observed to be strongly consistent with the course of the disease. Moreover, progression free survival analysis showed that patients experienced a significant early decrease of POSTN tended to obtain more benefits from the treatments.

**Conclusions:**

The current study suggests that soluble POSTN is an informative serum biomarker to complement the current clinical approaches for early and long-term chemotherapy efficacy monitoring in advanced BCa.

**Supplementary Information:**

The online version contains supplementary material available at 10.1186/s12935-024-03298-1.

## Introduction

Breast cancer (BCa) is the most diagnosed malignant disease worldwide and one of the leading cause of cancer death among women [1]. In China, the incidence and mortality rate of breast cancer has been increasing for decades [2]. Outcomes for patients with early-stage disease have been improved significantly in recent years, thanks to the advent of more effective systemic treatment options [3]. However, metastatic recurrence remains a significant problem for BCa patients, accounting for more than 90% of tumor-related deaths [4]. For patients with metastatic disease, the 5-year survival rate was less than 31% [5], compared to an average of about 90% on average for all BCa patients [3]. Improving the prognosis of metastatic BCa depends on well-timed and optimized treatment strategies.

Chemotherapy plays vital roles in the control of BCa. For the majority of patients in early stages, surgery remains the primary treatment, while neoadjuvant chemotherapy and/or adjuvant chemotherapy could significantly reduce the recurrence rate and correlate with long-term disease-free survival and overall survival [6]. However, for advanced patients, chemotherapy regimens are usually the primary treatment strategy. Fortunately, recent years have witnessed the expeditious development of pharmaceutical agents for the chemotherapy of BCa. However, clinically, due to the complexity of the disease, especially the cellular and molecular heterogeneity of tumors, the response of patients to a particular chemotherapy regimens varies from person to person [7]. Therefore, to guarantee the effectiveness of the chemotherapy, it is essential for guiding cancer therapy decisions that the response to treatment can be timely and accurately assessed.

The primary method currently used to evaluate the effectiveness of chemotherapy for breast cancer is radiography, which follows the Response Evaluation Criteria in Solid Tumors (RECIST) [8]. However, this approach has several practical limitations; it is often expensive, time-consuming, and can potentially lead to side effects from contrast agents. Tumor marker assays, on the other hand, do not share these drawbacks. Tumor markers, which encompass a broad range of categories including cells, proteins, and nucleic acids, can offer many benefits for cancer diagnosis and treatment, such as early detection, personalized treatments, reduced side effects, less invasive diagnostics, and improved quality of life [9]. Notably, the analysis of circulating nucleic acids, often termed ‘liquid biopsies’, serves to monitor treatment response, evaluate the emergence of drug resistance, and quantify minimal residual disease [10–12]. However, they also have some limitations, such as lack of specificity, sensitivity, and standardization, as well as the need for further validation and clinical trials [13]. Numerous efforts have been undertaken to introduce new biomarkers that could augment existing clinical strategies for monitoring chemotherapy efficacy in BCa [14].

In the past decade, liquid biopsies have received a great deal of attention [15]. Markers widely studied in liquid biopsies include circulating tumor cells (CTCs), circulating cell-free tumor DNA (ctDNA) and noncoding RNA (ncRNA) [15, 16]. With the increasing maturity of CTCs detection technology, its clinical value has received more and more attention [17]. However, only one assay, the CellSearch System, is currently in the transition phase from FDA approval to clinical application, and new assays still need to be validated in clinical trials to prove their efficiency [18]. As the release of tumor-associated DNA and RNA into blood is a common event in patients with cancer, quantitative evaluation of circulating nucleic acid may provide real-time information on genetic and epigenetic profiles associated with BCa development, progression, and response to therapy [19, 20], with superiority well beyond tissue biopsies, which can deliver a limited snapshot of the tumor lesions [15]. However, the challenge of standardizing operations across different technology platforms and controlling pre-analytical and analytical factors is still such a huge challenge that it has become a major impediment to their practical application [15, 21]. In contrast, traditional protein markers still have their own advantages today. One of the most obvious advantages, for instance, is the feasibility of developing commercial kits with standardized procedures for valuable marker candidates.

POSTN is a secreted extracellular matrix (ECM) glycoprotein, which was highly expressed in BCa tissues, and usually associated with poor clinical outcomes [22–25]. Our recently published work found that serum POSTN was a novel biomarker complementing CA15-3 and CEA for BCa diagnosis and metastasis prediction [26]. However, the clinical significance of circulating POSTN in BCa remains to be fully assessed. The aim of this work was to investigate whether soluble POSTN in circulating could be served as a potential biomarker for chemotherapy efficacy monitoring in BCa.

## Materials and methods

### Patients and specimens

This study was conducted at Tianjin Medical University Cancer Institute and Hospital between June 2016 and February 2022. All patients with BCa were confirmed by pathologic examination. Serum samples were harvested before surgery or treatment and were collected, aliquoted, and frozen at − 80 °C till use. To clarify the difference between preoperative and postoperative marker levels, blood samples were also collected from 15 BCa patients 2 weeks after surgery. The disease was staged according to the American Joint Committee on Cancer (AJCC) TNM (tumor–node–metastasis) classification. Tumor size was evaluated in patients without distant metastasis. Clinic characteristics of patients were summarized in Table 1.

**Table 1:** Correlation of serum POSTN levels and clinicopathological characteristics of BCa patients

Characteristics	Number	POSTN(ng/mL) Median (interquartile range)	*P* value
Age (median)	53.0		
≤ 60	168	23.49 (15.64–35.50)	
> 60	63	19.09 (13.12–31.59)	0.32^a^
Tumor size (maximum diameter)*			
≤ 2	46	14.93 (10.07–19.02)	
> 2& ≤ 5	69	17.99 (12.48–27.95)	
> 5	22	28.05 (19.56–42.79)	< 0.0001^b^
TNM stage			
I	31	16.11 (11.83–22.46)	
II	72	17.59 (10.14–25.38)	
III	34	21.01 (16.13–32.65)	
IV	94	26.77 (17.85–49.95)	< 0.0001^b^
Lymph node metastasis			
No	78	16.47 (11.06–24.31)	
Yes	153	24.72 (16.29–39.68)	< 0.0001^a^
Distant metastasis			
No	137	17.55 (12.26–26.45)	
Yes	94	26.77 (17.85–49.95)	< 0.0001^a^
Molecular subtype			
Luminal A	77	21.09 (12.76–31.85)	
Luminal B	81	19.67 (13.58–28.91)	
HER2-enriched	38	25.85 (13.72–50.08)	
Triple negative	35	18.24 (15.20–26.79)	0.59^b^
Chemotherapy efficacy monitoring			
Non-HER2-targeted	28	22.01 (15.46–30.85)	
HER2-targeted	30	24.05 (16.93–51.02)	0.43^a^
Response			
PR	33	24.61 (16.88–45.47)	
Non-PR	25	20.54 (15.67–27.02)	0.44^a^

^*^Tumor size was evaluated in patients without distant metastasis

^a^Wilcoxon rank-sum test

^b^Kruskal–Wallis test

In total, 231 patients with different stages of BCa was included in this study. Of those BCa patients, 58 patients with inoperable metastatic disease who received only chemotherapy during the observation period were enrolled for longitudinal evaluation. Previous malignancy other than BCa, incomplete observations, or radiotherapy during the observation period was considered as exclusion criteria. To compare the monitoring efficiency of POSTN for HER2-targeted therapy and non-targeted therapy, relatively more patients with HER2 positive status were included. Thirty of these patients received targeted therapy and the other 28 patients received non-targeted therapy. Serum samples from patients before the first cycle (C0, baseline), third cycle (C2) and fifth cycle (C4) of chemotherapy were obtained, aliquoted, and snap frozen at − 80 °C till use. Computed tomographic (CT) scans were typically performed within two weeks prior to the first cycle of chemotherapy (C0), and repeated with chemotherapy for treatment efficacy evaluation. Treatment responses at C4 were determined according to the Response Evaluation Criteria in Solid Tumors (RECIST) version 1.1 [27]. Radiographic responses were recorded as partial response (PR) if the maximum tumor diameters (MTD) decreased by at least 30%, as progressive disease (PD) if the MTD increased by at least 20% or if new lesions appeared, or as stable disease (SD) if neither criterion was reached. Of the 58 patients, 33 were observed with PR response, 21 with SD response, and 4 with PD response at time-point C4. Molecular subtype, chemotherapy regimens, and response at C4 were summarized in Additional file 1. Long-term chemotherapy efficacy monitoring were performed in four patients with complete observations with disease progression. Progression free survival within twenty-four months was defined as the time interval from partial response to disease progression.

### Measurement of serum POSTN

Soluble POSTN in patients and control subjects were measured by the ELISA method using a commercial kit (R&D System Inc., MN, USA), according to the instructions from the manufacturer, which has been described previously [28]. Briefly, 96-well ELISA micro-plates were coated overnight with POSTN capture antibody. After washing and incubating with blocking buffer, 100 μL of tenfold diluted serum samples were added. Following detection antibody and avidin-horseradish peroxidase-conjugated secondary antibody, the amount of bound conjugate was determined by adding the TMB substrate solution. The absorbance was measured at 450 nm using a Model 680 micro-plate reader (Bio-Rad, Hercules, CA). All samples were tested in duplicate.

### Measurement of serum CEA and CA15-3

Serum CA15-3 and CEA were detected with electrochemiluminescence immunoassays on the Roche Cobas E601 immunoassay analyzer (Roche Diagnostics, Mannheim, Germany) equipped with dedicated reagents, according to the instructions from the manufacturer. All of the assays were performed at the department of Laboratory Medicine, Tianjin Medical University Cancer Institute and Hospital, Tianjin.

### Statistical analysis

Statistical significance was determined with the Wilcoxon rank-sum tests (difference in marker levels between two groups), Kruskal–Wallis test (difference in marker levels among more than two groups), and Wilcoxon signed-rank tests (difference in marker levels between preoperative and postoperative). Agreement and relevance between radiographic response and markers’ response were assessed with Cohen’s weighted (Fleiss-Cohen weights) kappa coefficient, Wilcoxon rank-sum tests and Fisher Chi-square test. Nonparametric Spearman's correlation coefficients method was used to evaluate the association between serum marker and the MTD, as well as association between the baseline levels and the change rate of POSTN. Progression free survival was analyzed using the Kaplan–Meier product limit method and log-rank test. All of these statistical analyses were performed with SPSS 26 software (SPSS Inc., Chicago, IL, USA). Values of *P* ≤ 0.05 were considered statistically significant.

## Results

### Tumor burden-associated soluble POSTN level in BCa patients

**Fig. 1:**
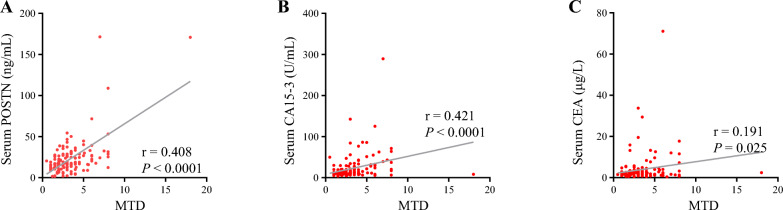
Scatter plots of POSTN, CA15-3 and CEA in prevalence assessment. **A**, the correlation between levels of POSTN and the corresponding MTD in BCa patients without distant metastasis (n = 137). **B**, the correlation between levels of CA15-3 and the corresponding MTD in BCa patients without distant metastasis (n = 137). **C**, the correlation between levels of CEA and the corresponding MTD of in BCa patients without distant metastasis (n = 137). *MTD* maximum tumor diameters

Serum levels of POSTN, as well as CA15-3 and CEA, were evaluated in all BCa. Compared with the recently published work [26], the correlation between the characteristics of BCa patients and POSTN levels was analyzed from a much larger cohort, particularly between its levels and tumor volume. As shown in Table 1, POSTN levels were significantly correlated with TNM stages, lymph node metastasis, distant metastasis of BCa patients, but not with age and molecular subtypes. Moreover, in patients without distant metastasis, a significant correlation between soluble POSTN levels and MTD was observed for the first time (Fig. 1A and Table 1). A similar trend with tumor volume was also observed in CA15-3 (Fig. 1B). As for CEA, its correlation with tumor burden of BCa was relatively weak (Fig. 1C). To further illustrate the relationship between the biomarkers and BCa, serum levels of POSTN, CA15-3 and CEA were compared in samples collected prior to and 2 weeks after surgery. The postoperative levels of three markers were decreased significantly (Additional file 2). The above results indicated that, in comparison with CA15-3 and CEA, POSTN showed promising potential as a novel biomarker for tumor burden assessment of BCa.

### Early dynamics of POSTN in BCa patients treated with chemotherapy regimens

**Fig. 2:**
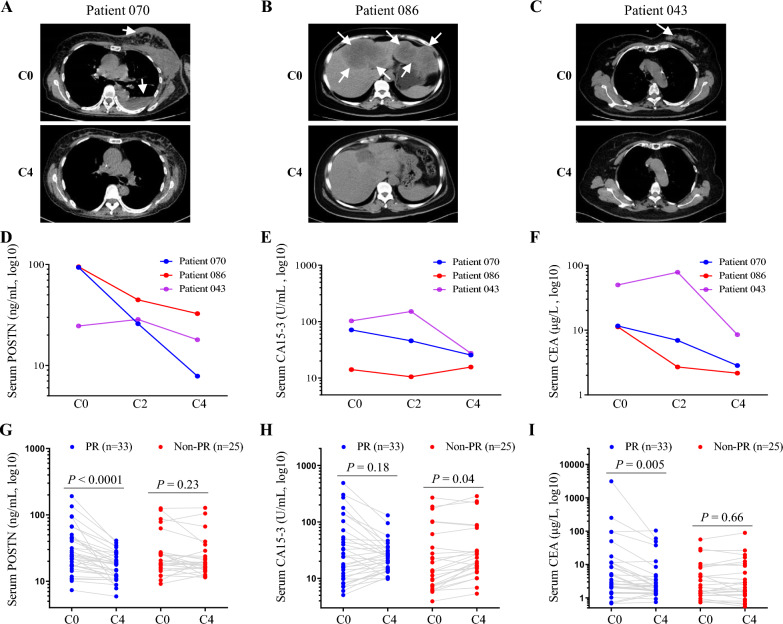
Early dynamics of markers in BCa patients treated with chemotherapy regimens. **A**–**C**, representative CT images of three partial-response cases at time-point of C0 and C4. Tumor lesions before and after the treatment were indicated by arrows. D-F, dynamics of POSTN (**D**), CA15-3 (**E**) and CEA (**F**) in the three representative cases at time-point of C0, C2 and C4. G-I, the dynamics of the POSTN (**G**), CA15-3 (**H**) and CEA (**I**) in longitudinal assessment of all 58 patients annotated with the tumor early response status to chemotherapy at C0 and C4. *PR* partial response (n = 33), *Non-PR* non-partial response (n = 25)

In the following study, we investigated whether longitudinal follow-up of soluble POSTN could recapitulate the early chemotherapy efficacy as assessed by imaging. Clinical data of 58 BCa patients with advanced disease receiving targeted or non-targeted chemotherapy regimens with determined treatment response were analyzed in our study. As shown in Table 1, no obvious difference in POSTN levels was observed in the two groups. Of those patients, 33 PR and 25 non-PR (21 SD and 4 PD) were determined according to the RECIST version 1.1 (Fig. 2A–C). Similarly, there was no significant difference in baseline POSTN levels in the PR and non-PR groups (Table 1). Levels of POSTN, CA15-3 and CEA in serum samples obtained at time-point of C0, C2 and C4 of chemotherapy were detected (Fig. 2D–F). Moreover, the dynamics of the three markers at different time-points in longitudinal samples from all 58 patients were annotated with the tumor early response status to chemotherapy, as shown in Additional file 3 (time-points C0, C2 and C4) and Fig. 2G–I (time-points C0 and C4). Figure 2G shows that the serum levels of POSTN in 33 PR patients were significantly reduced after treatment for 4 cycles (39.46 ± 39.40 vs. 18.50 ± 9.04, *P* < 0.0001), but no significant change was observed in 25 non-PR patients (33.99 ± 33.21 vs. 28.98 ± 29.03). In contrast, we did not observe significant changes of CA15-3 in 33 PR patients (Fig. 2H), while a little increasing in 25 non-PR patients (Fig. 2H, 45.60 ± 68.96 vs. 53.43 ± 76.73, *P* = 0.04). The changes of CEA somehow appeared to be similar to those of POSTN in both PR and non-PR patients, as shown in Fig. 2I.

### Performances of three markers in early chemotherapy efficacy monitoring of BCa

**Fig. 3:**
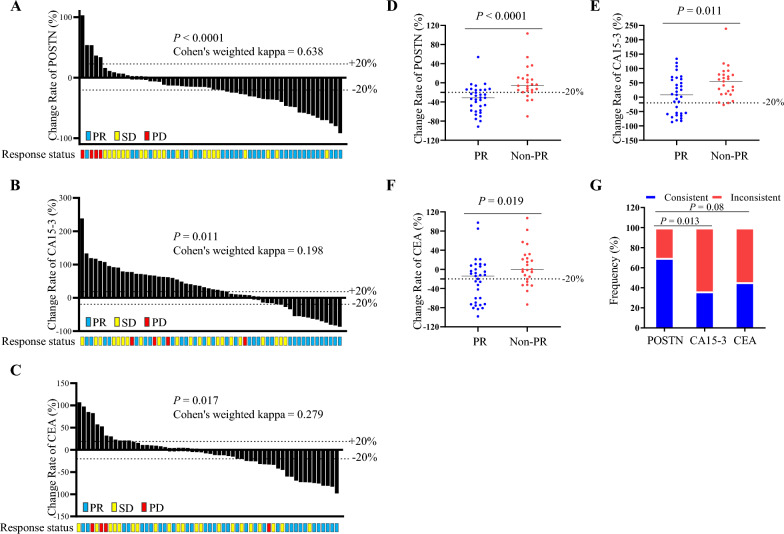
Performances of POSTN in early chemotherapy efficacy monitoring of BCa. **A**–**C**, agreement between radiographic response and markers’ response in 58 cases. Radiographic response was measured according to RECIST, version 1.1. Dotted lines indicated a 20% decrease or increase of markers. Agreement levels was evaluated by Cohen's weighted (Fleiss-Cohen weights) kappa coefficient. **D**–**F**, change rate of three markers was calculated by dividing the change level by the baseline level in cases with PR (n = 33) and Non-PR (n = 25) response. Dotted lines indicated decrease or increase of markers. **G**, comparison of consistent rate of three markers in cases with PR response. Dotted lines indicated a 20% decrease of markers. **H**, consistent frequency comparison among three markers in cases with partial response. *PR* partial response, *SD* stable disease, *PD* progressive disease

To evaluate the performances of three markers in early chemotherapy efficacy monitoring of BCa patients, agreement between radiographic response and markers’ response were assessed with Cohen’s weighted kappa coefficient, and 20% is considered to be the dividing line between significant and limited change: an increase of more than 20% corresponded to PD; a decrease of more than 20% corresponded to PR, while changes less than 20% corresponded to SD. As shown in Fig. 3A–C**,** POSTN was observed with the best performance among the three markers (kappa of POSTN = 0.638, *P* < 0.0001; kappa of CA15-3 = 0.198, *P* = 0.011; kappa of CEA = 0.279, *P* = 0.017). In addition, although alterations of three markers were associated with clinical benefit of partial response evaluated by imaging, the median alteration of POSTN in patient with PR response was (− 31.2%, Fig. 3D) much more pronounced than that of CA15-3 (8.0%, Fig. 3E) and CEA (− 14.0%, Fig. 3F). Next, we further compared the agreement rates of the three markers in recapitulating PR response. As shown in Fig. 3G**,** if a decrease of more than 20% was considered as consistent, decline of POSTN was observed in 69.7% (23 out of 33) of PR patients, which was higher than that of CA15-3 (36.4%) and CEA (45.5%), and even their combination (63.6%). While the combined performance of the three indicators was optimal, achieving a consistency rate as high as 93.9% (31 out of 33, Additional file 4).

**Fig. 4:**
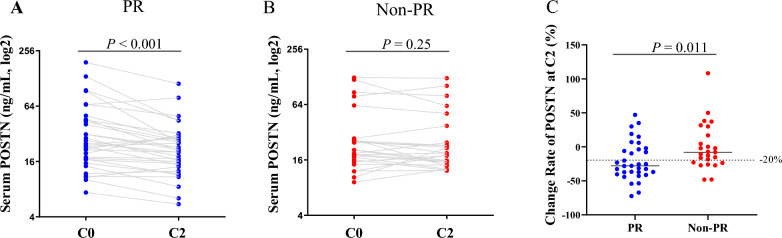
Performance of alterations of POSTN as early as C2 in early chemotherapy efficacy monitoring of BCa. **A**, **B**, POSTN levels at time-point of C0 and C2 in cases with in partial response (**A**) and non-partial response (**B**). **C**, change rate of POSTN at C2 was calculated by dividing the change level by the baseline level in cases with PR (n = 33) and Non-PR (n = 25) response

Twenty four out of 30 patients treated with HER2-targeted therapy achieved a PR response, which was significantly better than those treated with non-HER2-targeted therapy (9 out of 28 patients) as shown in Additional file 1. However, no obvious difference was observed in the monitoring performance of POSTN in PR patients treated with targeted or non-targeted chemotherapy (Additional file 5). The response of soluble POSTN at C2 was also investigated, which were also associated with the early efficacy of chemotherapy for PR patients (Fig. 4A–C). These results indicate that serum POSTN could be a potential marker for the early monitoring of chemotherapy efficacy, with superiority well beyond CA15-3 and CEA.

### Effects of baseline levels of POSTN on its early monitoring performance

**Fig. 5:**
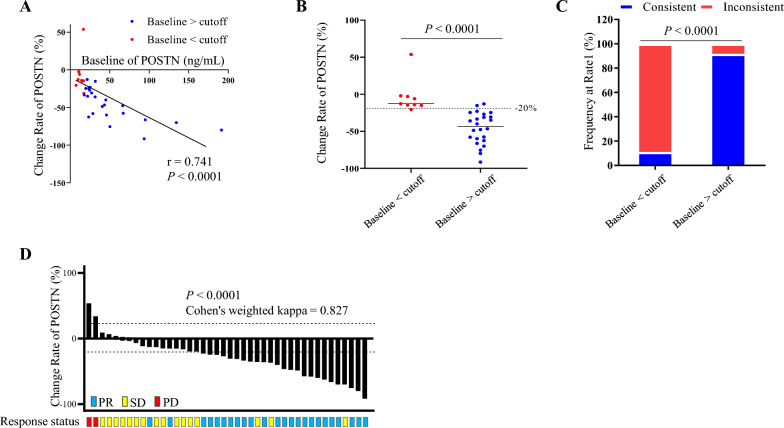
Effects of baseline levels of POSTN on its early monitoring performance. A, the correlation between baseline levels of POSTN and its change rate in cases with partial response (n = 33). **B**, comparison of change rate of POSTN in partial response cases with baseline above (n = 24) and below (n = 9) the cutoff value. **C**, comparison of consistent rate of POSTN in cases with partial response cases with baseline above and below the cutoff value. Dotted lines indicated a 20% decrease of markers. **D**, agreement between radiographic response and POSTN’s response in 42 cases with baseline above the cutoff value

Although a relatively high agreement was observed in cases with PR response, alterations of soluble POSTN in a minority of cases were too small to recapitulate the response. Further analysis was performed to explore the underlying reasons. Impressively, the baseline levels of POSTN were found to be significantly correlated with the change rates of POSTN in the patients with PR response (Spearman r = 0.741, *P* < 0.0001, Fig. 5A). As shown in Table 1, levels of POSTN have significant individual variations in BCa patients, and even among patients with advanced disease, there was still a proportion of patients with fairly low levels of POSTN. To separate this group of patients, the median level of POSTN (17.19 ng/mL) in patients with disease at stage I and II, which could both indicate the presence of tumor lesions and be applicable to patients with a low tumor burden, was established as the cutoff value. Thus, cases with PR response could be divided into two groups according to its baseline levels of POSTN. Remarkably, patients with high baseline levels were observed with much higher change rates of POSTN (− 3.80 ± 22.45 vs. − 45.77 ± 21.42, *P* < 0.0001, Fig. 5B) and agreement rates (91.7% vs. 11.1%, *P* < 0.0001, Fig. 5C) in recapitulating PR response. Moreover, with the remove of those cases with low baseline levels, the agreement between radiographic response and POSTN response was significantly improved. In such a situation, the weighted kappa was equal to 0.827, which represents a good strength of agreement (Fig. 5D, *P* < 0.0001).

### Performance of POSTN in long-term chemotherapy efficacy monitoring of BCa patients

**Fig. 6:**
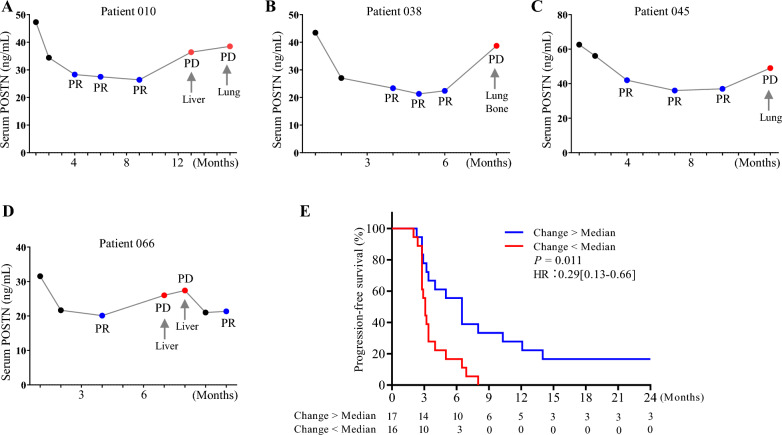
Performance of POSTN in long-term chemotherapy efficacy monitoring of BCa patients. **A**–**D**, dynamics of POSTN in four patients with complete observations with disease progression. **E**, progression free survival analysis was performed in patients with change above (n = 17) and below (n = 16) the median levels. *PD* progressive disease

Long-term chemotherapy efficacy monitoring were performed in four patients with complete observations with disease progression. A strong consistency of POSTN response was observed in all four cases (patient 010, 038, 045 and 066) with progressive disease in long-term follow-up (Fig. 6A–D). In contrast, much weaker consistency were observed in the response of CA15-3 and CEA (patient 010, 038 and 066, Additional file 6). Finally, we tried to assess if PR patients with significant decrease of POSTN at C4 would obtain more benefits from the treatments. The association between POSTN response and progression free survival outcomes was evaluated. We found that PR patients with more decrease of POSTN (change above median) at both time-point of C4 (Fig. 6E, *P* = 0.011) and C2 (Additional file 7, *P* = 0.039) were associated with significantly long progression free survival.

## Discussion

The current approach to determining chemotherapy efficacy in BCa through radiography presents several limitations, including high costs, time consumption, and potential side effects from contrast agents [29]. Furthermore, the blood markers CA15-3 and CEA, which are commonly used in BCa management, have shown unsatisfactory performance due to their limited sensitivity and specificity [30]. Our study has demonstrated a significant association between soluble POSTN levels and both tumor volume and TNM stages in BCa patients without distant metastasis. Importantly, the dynamics of soluble POSTN were found to effectively reflect both early and long-term chemotherapy efficacy, as determined by imaging. This suggests that soluble POSTN could serve as a valuable tool in BCa management, complementing existing methods and potentially enhancing treatment outcomes.

To the best of our knowledge, this is the first study to explore the potential of soluble POSTN in monitoring chemotherapy efficacy, particularly in patients with advanced BCa. Our findings suggest that the dynamics of soluble POSTN outperform those of CA15-3 and CEA in reflecting early chemotherapy efficacy as assessed by imaging. While previous studies have proposed that CEA and CA15-3 may serve as useful biomarkers for predicting therapeutic response in advanced BCa patients [31]. However, ASCO has cautioned that levels of CEA and CA15-3 should be interpreted carefully during the initial 4–6 weeks of a new therapy due to the possibility of spurious increases [32–34]. In contrast, our study indicates that POSTN is less susceptible to this issue. Our results show that a greater number of patients with a PR exhibited elevated levels of CA153 and CEA compared to POSTN. POSTN is highly regulated in cancer-associated stromal cells, suggesting that signals from tumor cells, such as TGF-β, may primarily trigger POSTN expression in the cancer-associated ECM [35–38]. Therefore, chemotherapy-induced apoptosis or necrosis of tumor cells in the initial cycles may lead to a significant increase in serum CA15-3 and CEA levels, while POSTN may have already been degraded during the detection window period. Furthermore, the production of POSTN by cancer-associated stromal cells may be further reduced due to fewer maintenance signals from tumor cells. As a result, levels of soluble POSTN may provide a more accurate real-time snapshot of tumor burden.

Early evaluation of therapeutic efficacy is crucial in reducing the mortality rate of BCa. Noninvasive biomarkers, which provide timely and accurate reflections of the disease state, are of primary concern. In particular, for patients with non-responsive tumors, early insight into treatment outcomes can be invaluable for treatment reorientation. However, it’s important to note that the efficiency of POSTN in reflecting early chemotherapy responses, especially partial responses, varies with its baseline level. Not all BCa patients exhibit elevated serum POSTN levels. In fact, a subset of patients with advanced disease display relatively low POSTN levels. This suggests significant individual differences in the levels of protein markers, such as POSTN, CA153, and CEA, among BCa patients and even normal subjects. Unlike other liquid biopsy targets, such as ctDNA, protein markers need to be expressed before they can indicate the presence of tumors. However, their successful expression in a dysfunctional environment is often uncertain. This limitation of protein markers should be taken into account when interpreting their levels in the context of disease monitoring and treatment response.

Our study has several limitations that warrant discussion. Firstly, the relatively small sample size may limit the generalizability of our findings. Although previous research has found a correlation between mRNA levels of POSTN and the status of HER2 [26], our study did not observe significant differences in serum POSTN among various BCa subtypes. Similarly, we did not find noticeable differences in POSTN dynamics between patients treated with conventional and targeted chemotherapy regimens. These findings suggest that the clinical significance of soluble POSTN in different BCa subtypes requires further exploration in a well-controlled study with a larger sample size. Secondly, long-term chemotherapy efficacy monitoring was performed on only four patients, which may have resulted in a potentially inadequate performance assessment of POSTN. Previous reports have suggested that POSTN plays a role in the induction process of BCa chemo-resistance [39, 40]. To fully understand the potential clinical implications of POSTN response in chemo-resistance or recurrence of BCa, a long-term follow-up of a larger cohort is currently underway. This will provide a more comprehensive insight into the potential therapeutic monitoring significance of soluble POSTN.

## Conclusions

To avoid inappropriately discontinuing potentially effective treatments or proceeding with ineffective therapy regimens, performing soluble POSTN analysis to assess early response to chemotherapy may be beneficial to patients. It’s important to note that tumor markers like soluble POSTN are not intended to replace RECIST, but rather to complement it by providing additional information on tumor biology and prognosis. Taken together, the current study suggest that soluble POSTN is an informative serum biomarker to complement the current clinical approaches for chemotherapy efficacy monitoring in BCa.

### Supplementary Information

**Additional file 1:** Molecular subtype, chemotherapy regimens, and response in chemotherapy efficacy monitoring.

**Additional file 2: **Comparison of preoperative and postoperative levels of three markers in serum of BCa patients. Representation of individual markers, A: POSTN, B: CA15-3, C: CEA. n = 15.

**Additional file 3: **Dynamics of the three markers in longitudinal cases at time-points C0, C2 and C4. Dynamics of the three markers in longitudinal cases annotated with the tumor early response status to chemotherapy at time-points C0, C2 and C4. Representation of individual markers, A: POSTN, B: CA15-3, C: CEA. n = 58.

**Additional file 4: **This paper presents a consistent frequency comparison of POSTN, CA15-3, and CEA, both individually and in combination, in cases with a partial response. The combination of CA15-3 and CEA, as well as the combination of three markers, is considered consistent as long as at least one marker exhibits a consistent change.

**Additional file 5: **Monitoring performance comparison of POSTN in partial response cases treated with targeted or non-targeted chemotherapy. A, change rate of POSTN was calculated by dividing the change level by the baseline level in partial response cases treated with targeted or non-targeted chemotherapy. B, change rate comparison in partial response cases treated with targeted or non-targeted chemotherapy.

**Additional file 6: **Dynamics of CA15-3 and CEA in cases with complete observations with disease progression. A-C, dynamics of CA15-3 in three patients with complete observations with disease progression. D-F, dynamics of CEA in three patients with complete observations with disease progression.

**Additional file 7: **Alterations of POSTN at C2 were associated with the progression free survival of cases with partial response. Progression free survival analysis was performed in patients with change of POSTN at C2 above and below the median levels.

## Data Availability

All data and other items supporting the results in the paper are available. If anyone would like to access these data, please contact the corresponding author Dong Dong (youxiudongdong@163.com) with a request.
