# Supplementary material for: Soluble Periostin is a potential surveillance biomarker for early and long-term response to chemotherapy in advanced breast cancer
Source: Cancer Cell Int. 2024 Mar 19;24:109. doi: 10.1186/s12935-024-03298-1 (PMC10953259; doi:10.1186/s12935-024-03298-1)
Supplement: Supplementary file 1 — Additional file 1: Molecular subtype, chemotherapy regimens, and response in chemotherapy efficacy monitoring. [file 12935_2024_3298_MOESM1_ESM.docx]

| **Patient ID** | **Molecular subtype** | **Chemotherapy Regimens** | **Treatment Category** | **Response** |
| --- | --- | --- | --- | --- |
| 045 | Luminal B | AC | Non-HER2-targeted | PR |
| 020 | Luminal B | AC | Non-HER2-targeted | PD |
| 009 | Luminal B | AC | Non-HER2-targeted | PR |
| 013 | Triple negative | AC | Non-HER2-targeted | SD |
| 015 | Luminal A | AC | Non-HER2-targeted | SD |
| 112 | Luminal A | AC | Non-HER2-targeted | SD |
| 016 | Luminal A | AC | Non-HER2-targeted | SD |
| 025 | Luminal A | AC-T | Non-HER2-targeted | SD |
| 026 | Luminal A | AC-T | Non-HER2-targeted | SD |
| 021 | Luminal A | EC | Non-HER2-targeted | SD |
| 022 | Luminal B | NP | Non-HER2-targeted | SD |
| 012 | Luminal A | NP | Non-HER2-targeted | SD |
| 035 | Triple negative | NX | Non-HER2-targeted | SD |
| 004 | Luminal A | TA | Non-HER2-targeted | PR |
| 017 | Luminal A | TA | Non-HER2-targeted | PR |
| 038 | Luminal A | TA | Non-HER2-targeted | PR |
| 019 | Luminal A | TE | Non-HER2-targeted | SD |
| 113 | Triple negative | TE | Non-HER2-targeted | PR |
| 010 | Triple negative | TP | Non-HER2-targeted | PR |
| 031 | Luminal B | TP | Non-HER2-targeted | PR |
| 032 | Luminal B | TP | Non-HER2-targeted | PR |
| 041 | Luminal B | TP | Non-HER2-targeted | SD |
| 044 | Triple negative | TP | Non-HER2-targeted | SD |
| 027 | Triple negative | TP | Non-HER2-targeted | SD |
| 005 | Luminal B | TX | Non-HER2-targeted | PD |
| 008 | Luminal B | TX | Non-HER2-targeted | PD |
| 030 | Luminal A | TX | Non-HER2-targeted | PD |
| 114 | Luminal B | Anastrozole + CDK4/6 inhibitor + Goserelin | Non-HER2-targeted | SD |
| 024 | Luminal B | TCbHP | HER2-targeted | PR |
| 102 | HER2-enriched | TCbHP | HER2-targeted | PR |
| 061 | HER2-enriched | TH | HER2-targeted | PR |
| 082 | HER2-enriched + Luminal B***** | TH | HER2-targeted | PR |
| 037 | HER2-enriched | THP | HER2-targeted | PR |
| 034 | HER2-enriched | THP | HER2-targeted | PR |
| 014 | HER2-enriched | THP | HER2-targeted | PR |
| 047 | Luminal B | THP | HER2-targeted | PR |
| 018 | Luminal B | THP | HER2-targeted | SD |
| 099 | HER2-enriched | THP | HER2-targeted | PR |
| 104 | HER2-enriched | THP | HER2-targeted | PR |
| 097 | HER2-enriched | THP | HER2-targeted | SD |
| 084 | HER2-enriched | THP | HER2-targeted | SD |
| 066 | HER2-enriched | THP | HER2-targeted | PR |
| 085 | HER2-enriched | THP | HER2-targeted | PR |
| 072 | HER2-enriched | THP | HER2-targeted | SD |
| 111 | HER2-enriched | THP | HER2-targeted | PR |
| 059 | HER2-enriched | THP | HER2-targeted | PR |
| 070 | HER2-enriched | THP | HER2-targeted | PR |
| 023 | Luminal B | THP | HER2-targeted | SD |
| 056 | Luminal B | THP | HER2-targeted | PR |
| 062 | Luminal B | THP | HER2-targeted | PR |
| 043 | Luminal B | THP | HER2-targeted | PR |
| 092 | Luminal B | THP | HER2-targeted | PR |
| 098 | Luminal B | THP | HER2-targeted | PR |
| 103 | Luminal B | THP | HER2-targeted | SD |
| 063 | Luminal B | Pyrotinib + Vinorelbine + Carboplatin | HER2-targeted | PR |
| 087 | Luminal B | Pyrotinib + Trastuzumab + Docetaxel + Cisplatin | HER2-targeted | PR |
| 086 | HER2-enriched | Pyrotinib + Vinorelbine + Capecitabine | HER2-targeted | PR |
| 091 | HER2-enriched | Pyrotinib + Trastuzumab + [Paclitaxel-albumin](file:///D:/%E7%99%BE%E5%BA%A6%E7%BF%BB%E8%AF%91/baidu-translate-client/resources/app.asar/app.html#/#) | HER2-targeted | PR |

*****, intraductal carcinoma, HER2-enriched; infiltrating area, Luminal B.

**Chemotherapy regimens (Non-HER2-targeted)**

**AC**: Cyclophosphamide + Anthracyclines; **AC-T**: Cyclophosphamide + Anthracyclines + Taxol; **EC**: Epirubicin + Cyclophosphamide; **NP**: Vinorelbine + Cisplatin; **NX**: Vinorelbine + Xeloda; **TA**: Taxol + Anthracyclines; **TE**: Pirubicin + Docetaxel; **TP**: Taxol + Carboplatin; **TX**: Docetaxel + Xeloda.

**Chemotherapy regimens (HER2-targeted)**

**TCbHP**: Trastuzumab + Pertuzumab + Docetaxel + Carboplatin; **TH**: Trastuzumab + [Paclitaxel-albumin](file:///D:/%E7%99%BE%E5%BA%A6%E7%BF%BB%E8%AF%91/baidu-translate-client/resources/app.asar/app.html#/#); **THP**: Trastuzumab + Pertuzumab + Docetaxel.
