# Supplementary figures and images for: Soluble Periostin is a potential surveillance biomarker for early and long-term response to chemotherapy in advanced breast cancer
Source: Cancer Cell Int. 2024 Mar 19;24:109. doi: 10.1186/s12935-024-03298-1 (PMC10953259; doi:10.1186/s12935-024-03298-1)

**A**

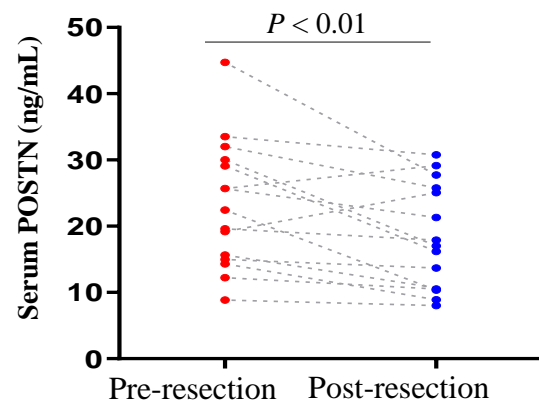

**B**

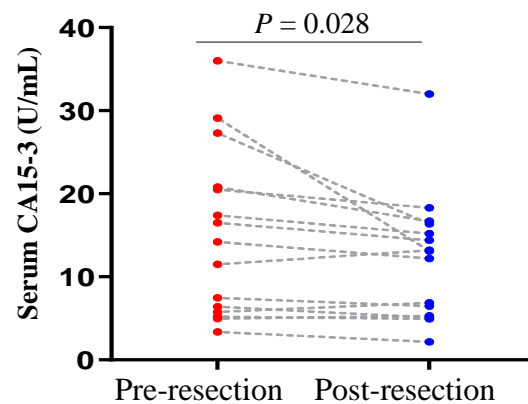

**C**

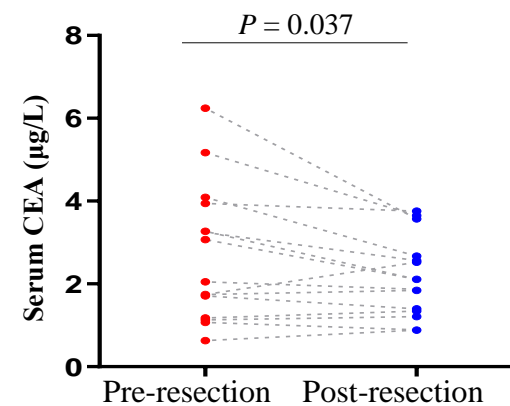

**A**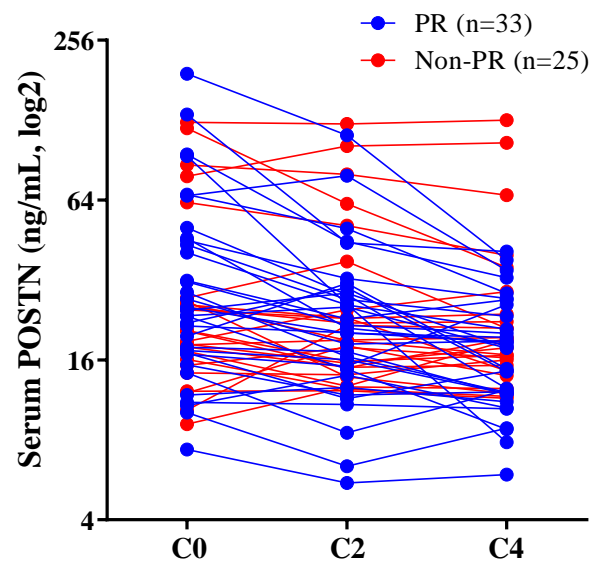**B**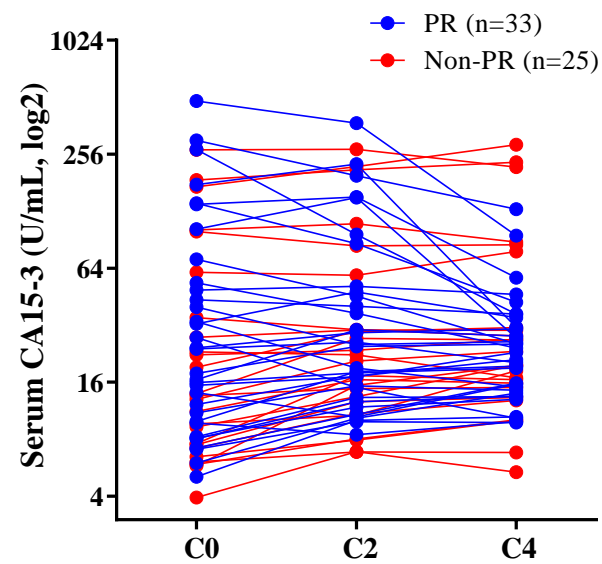**C**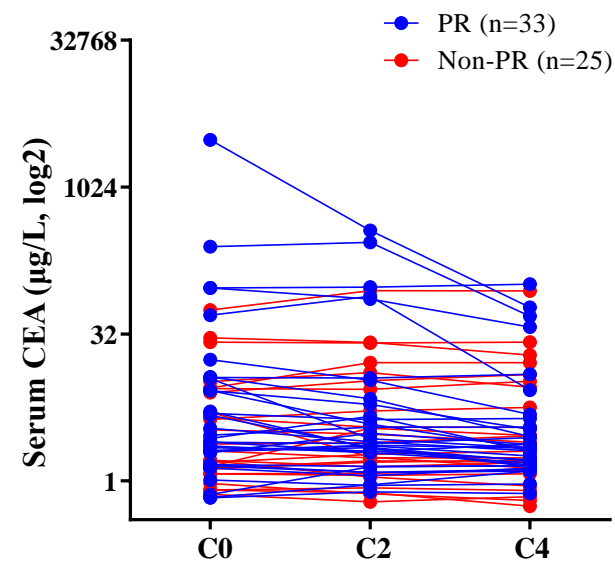

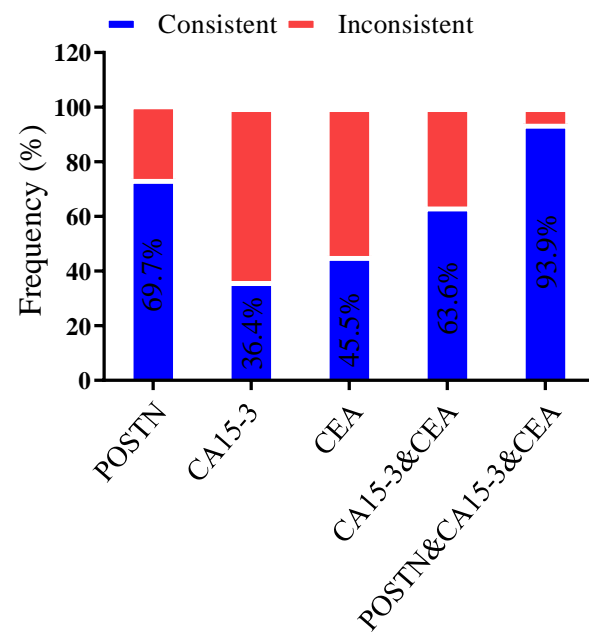

**A**

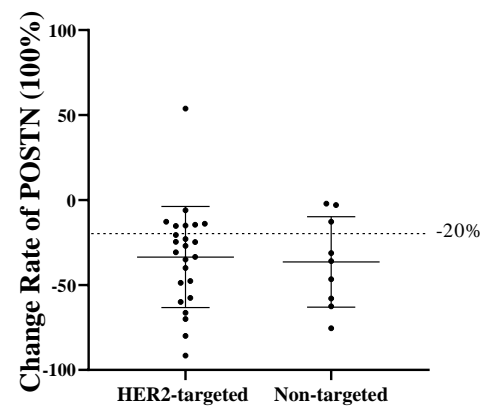

**B**

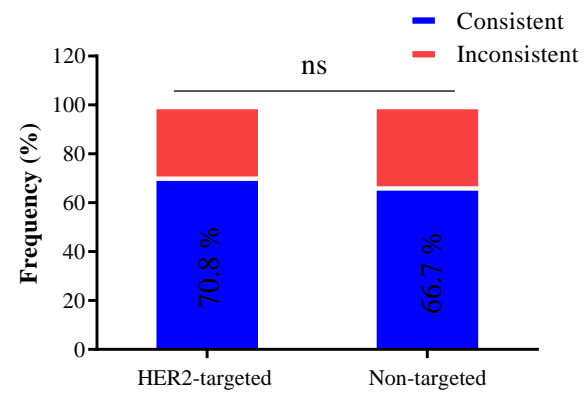

**A**

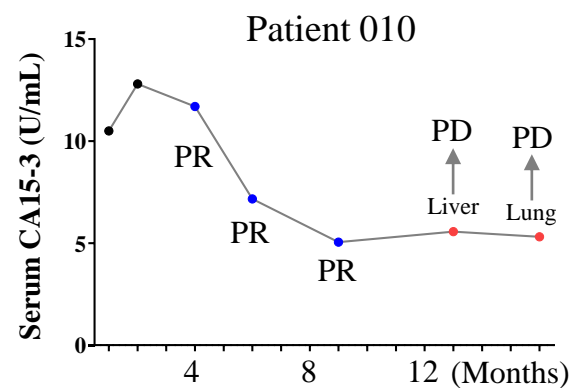

**B**

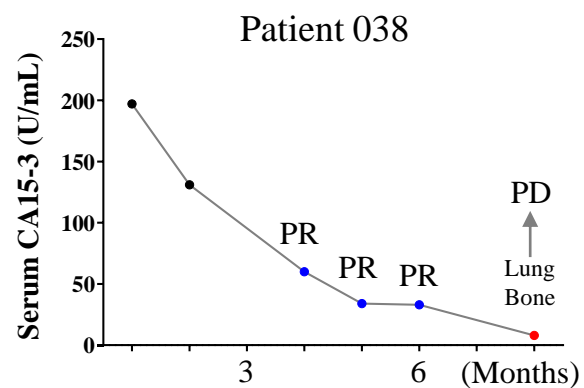

**C**

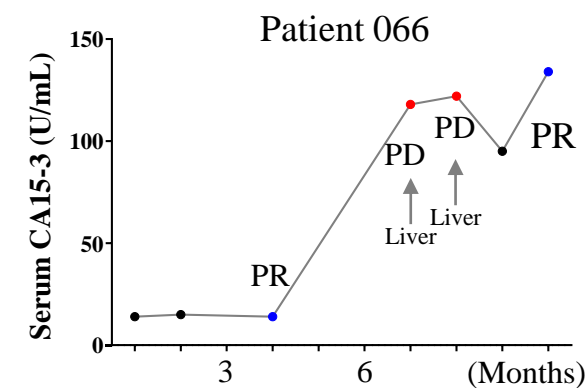

**D**

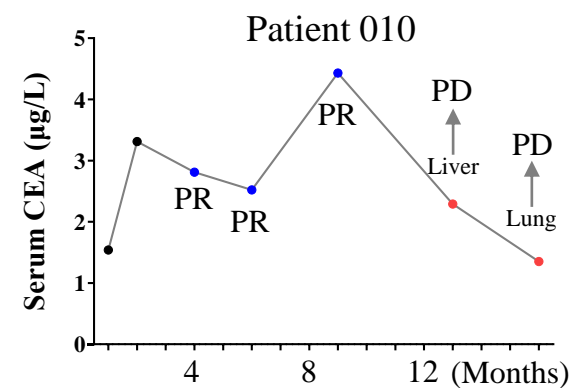

**E**

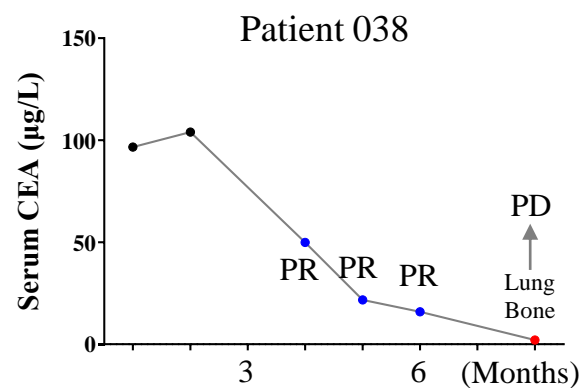

**F**

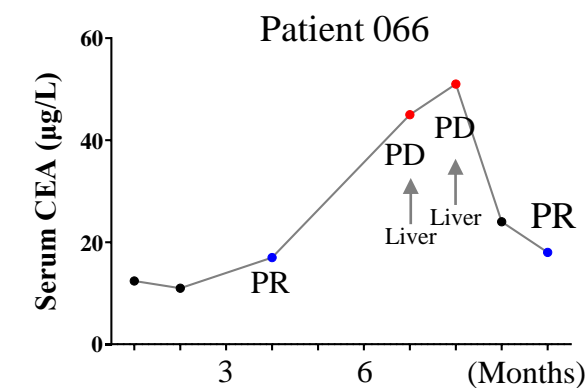

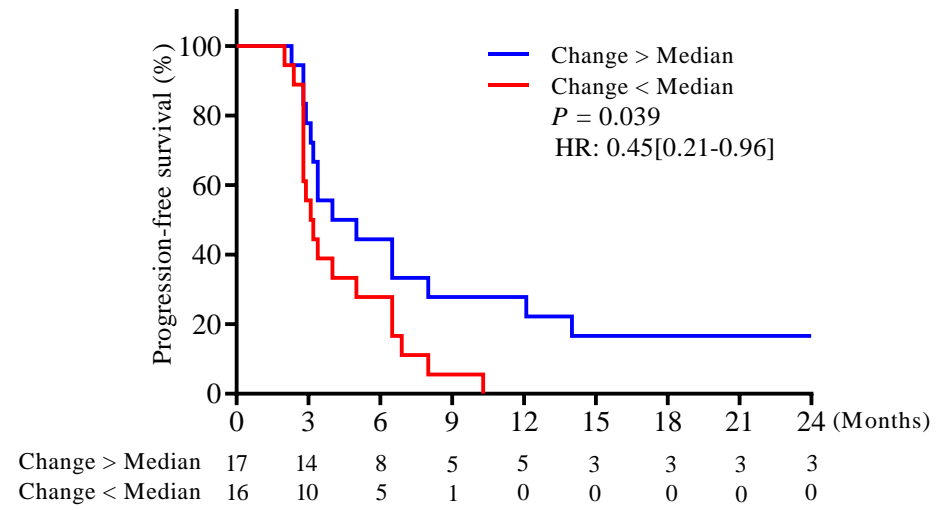

Supplement: Supplementary file 2 — Additional file 2: Comparison of preoperative and postoperative levels of three markers in serum of BCa patients. Representation of individual markers, A: POSTN, B: CA15-3, C: CEA. n = 15. [file 12935_2024_3298_MOESM2_ESM.pdf]

**A**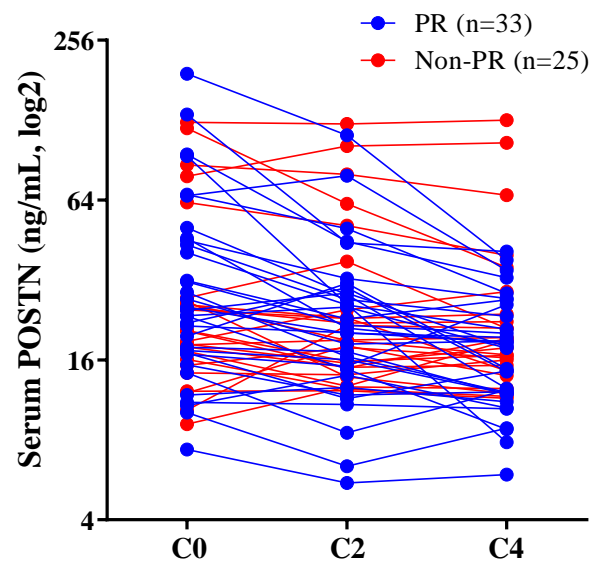**B**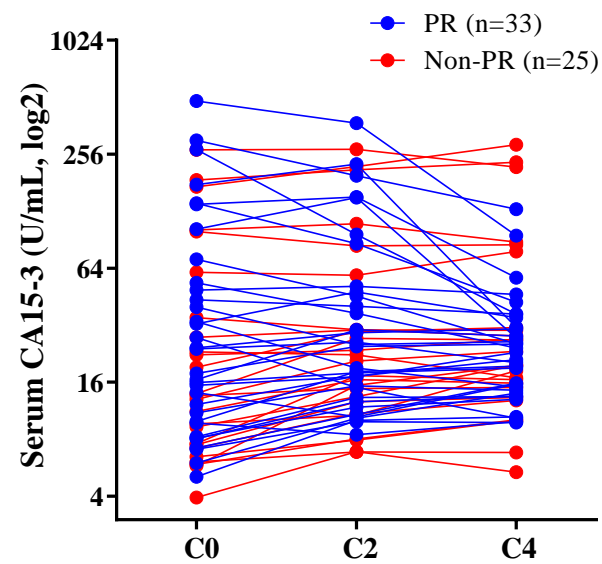**C**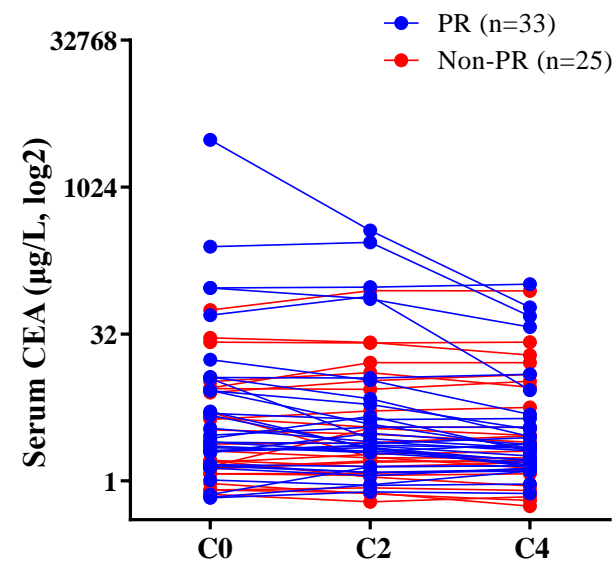

Supplement: Supplementary file 3 — Additional file 3: Dynamics of the three markers in longitudinal cases at time-points C0, C2 and C4. Dynamics of the three markers in longitudinal cases annotated with the tumor early response status to chemotherapy at time-points C0, C2 and C4. Representation of individual markers, A: POSTN, B: CA15-3, C: CEA. n = 58. [file 12935_2024_3298_MOESM3_ESM.pdf]

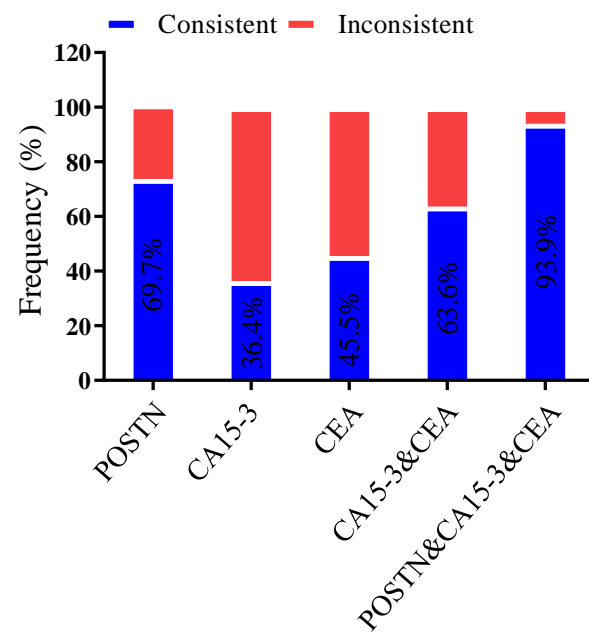

Supplement: Supplementary file 4 — Additional file 4: This paper presents a consistent frequency comparison of POSTN, CA15-3, and CEA, both individually and in combination, in cases with a partial response. The combination of CA15-3 and CEA, as well as the combination of three markers, is considered consistent as long as at least one marker exhibits a consistent change. [file 12935_2024_3298_MOESM4_ESM.pdf]

**A**

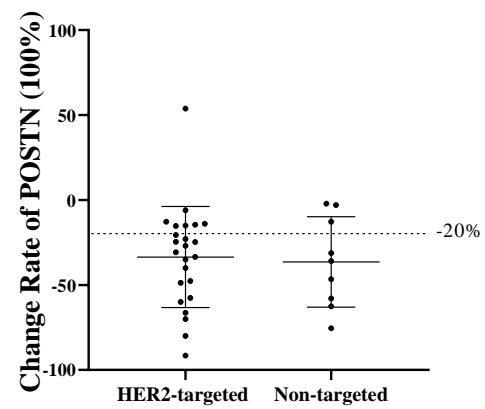

**B**

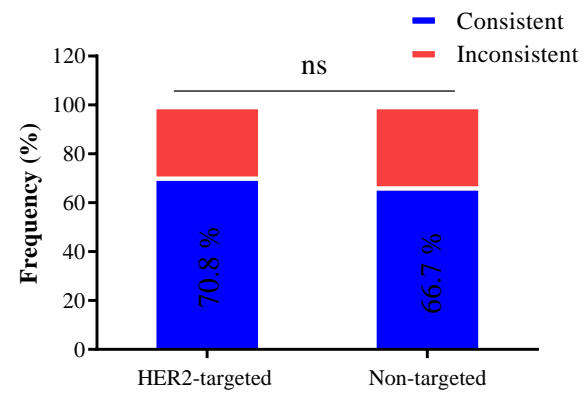

Supplement: Supplementary file 5 — Additional file 5: Monitoring performance comparison of POSTN in partial response cases treated with targeted or non-targeted chemotherapy. A, change rate of POSTN was calculated by dividing the change level by the baseline level in partial response cases treated with targeted or non-targeted chemotherapy. B, change rate comparison in partial response cases treated with targeted or non-targeted chemotherapy. [file 12935_2024_3298_MOESM5_ESM.pdf]

**A**

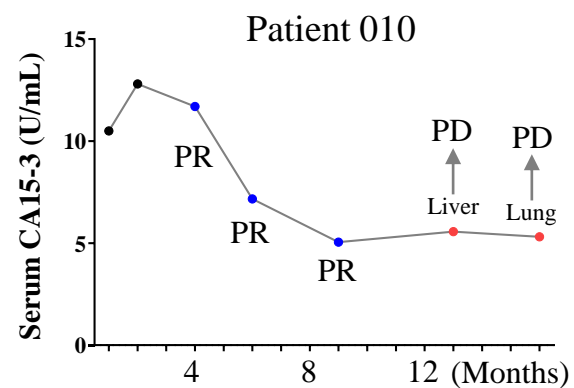

**B**

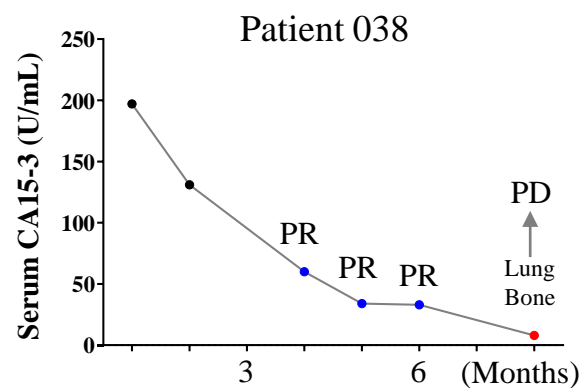

**C**

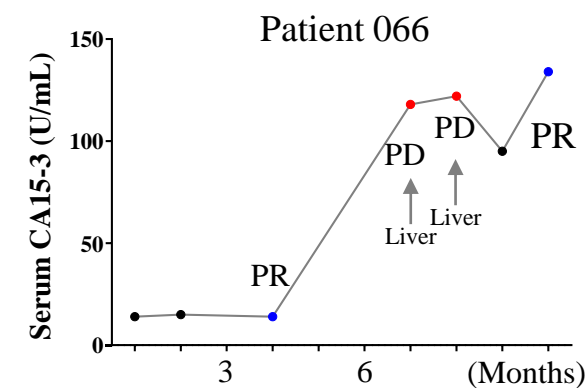

**D**

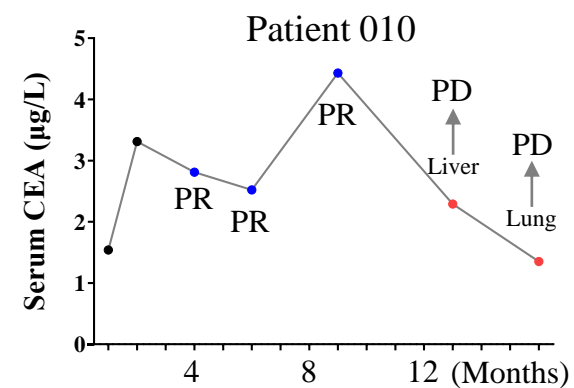

**E**

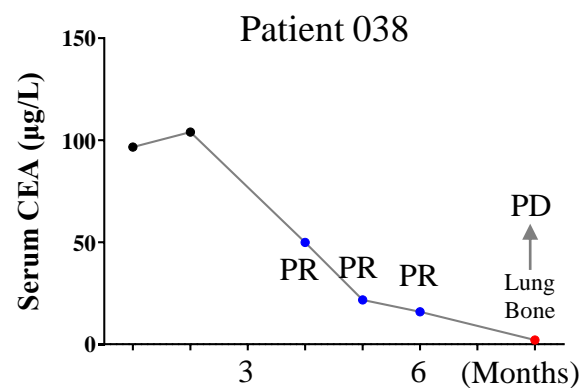

**F**

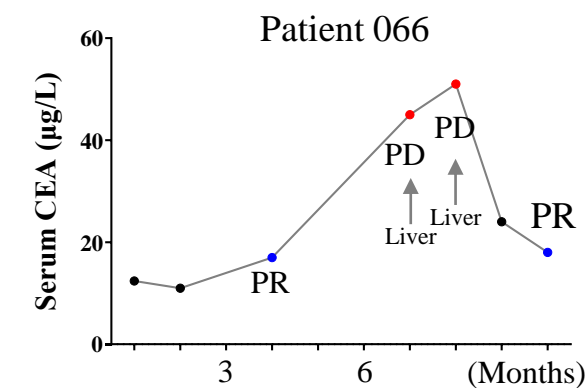

Supplement: Supplementary file 6 — Additional file 6: Dynamics of CA15-3 and CEA in cases with complete observations with disease progression. A-C, dynamics of CA15-3 in three patients with complete observations with disease progression. D-F, dynamics of CEA in three patients with complete observations with disease progression. [file 12935_2024_3298_MOESM6_ESM.pdf]

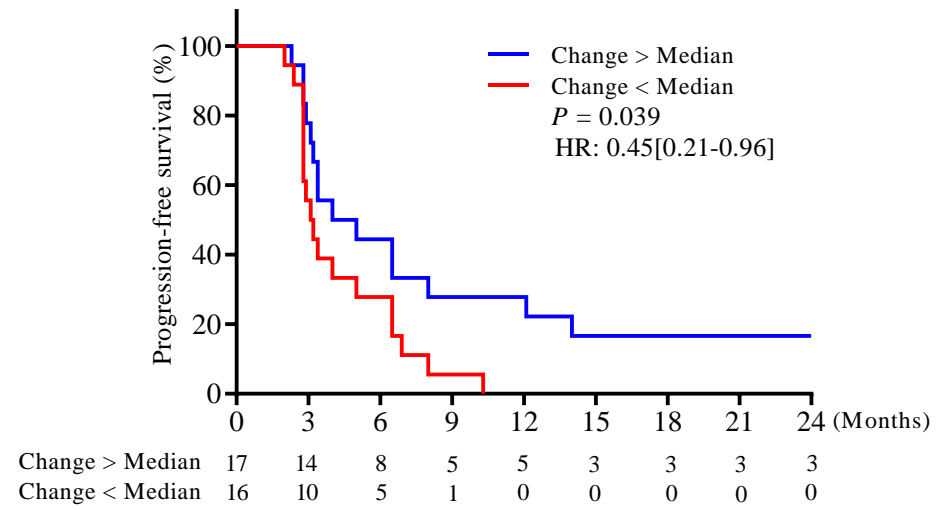

Supplement: Supplementary file 7 — Additional file 7: Alterations of POSTN at C2 were associated with the progression free survival of cases with partial response. Progression free survival analysis was performed in patients with change of POSTN at C2 above and below the median levels. [file 12935_2024_3298_MOESM7_ESM.pdf]
